# Re: Association between enuresis and obesity in children with primary monosymptomatic nocturnal enuresis

**DOI:** 10.1590/S1677-5538.IBJU.2019.0489

**Published:** 2020-01-10

**Authors:** Rujittika Mungmunpuntipantip, Viroj Wiwanitkit

**Affiliations:** 1 26 Medical Center Bangkok Thailand 26 Medical Center, Bangkok Thailand;; 2 DY Patil University Pune India Honorary professor, Dr. DY Patil University, Pune, India

To the editor,

We read the publication on “Association between enuresis and obesity in children with primary monosymptomatic nocturnal enuresis” with a great interest. Ma et al. concluded that “Obesity is associated with severe enuresis and low efficacy of behavioral therapy in children with nocturnal enuresis (1).” This report is concordant with a recent report from China (2) and Denmark (3). Nevertheless, the association between enuresis and obesity might be affected by some confounding personal illnesses. For example, the children with underlying hemoglobin disorder, such as thalassemia and sickle cell, has a chance for developing enuresis regardless of obesity (4). Indeed, those children with underlying hemoglobin disorder usually have malnutrition problem, not obesity (5).
